# Anti-inflammatory and barrier repair mechanisms of active components in *Daemonorops draco* Bl. for UVB-induced skin damage

**DOI:** 10.1038/s41598-025-01289-4

**Published:** 2025-05-17

**Authors:** Xingyi Wu, Ying Zhang, Fan Yi, Zaijun Geng, Miaomiao Guo, Xiao Ling, Jun Li, Li Li

**Affiliations:** 1 Beijing Key Lab of Plant Resource Research and Development, Beijing Technology and Business University, Fucheng Road, Haidian District, Beijing, 100048 China; 2 Beijing Lan Divine Technology Co. LTD, Culture Building, No. A59, Zhongguancun Street, Haidian District, Beijing, 100872 China; 3 School of Chinese Materia Medica, Modern Research Center for Traditional Chinese Medicine, Beijing University of Chinese Medicine, No. 11, North Third Ring East Road, Chaoyang District, Beijing, 100029 China

**Keywords:** *Daemonorops draco* Bl., (2S)-5-methoxy-6-methylflavan-7-ol (XJ-2), HaCaT, NF-κB, Anti-inflammation, Skin barrier, Cell biology, Drug discovery, Molecular biology

## Abstract

*Daemonorops draco* Bl. extract and its active ingredients can remove blood stasis and promote muscle and wound healing and are widely used in skin health and other fields. Modern pharmacological studies have demonstrated that this extract exerts excellent anti-inflammatory effects beneficial for skin barrier repair. However, the mechanism of action and monomeric components of *D. draco* remain unclear. Seven active monomers (XJ-1 ~ XJ-7) were extracted and purified from *D. draco*. The successful construction of the HaCaT inflammation model was achieved through the detection of IL-1β and TNF-α expressions in UVB-irradiated HaCaT cells. Based on this cellular model, (2 S)-5-methoxy-6-methylflavan-7-ol (XJ-2) was determined to be the best-screened monomer. The effects of XJ-2 on the production of reactive oxygen species (ROS) and Ca^2+^ in HaCaT cells were investigated using fluorescent probes and flow cytometry, respectively. The impact of XJ-2 on the expression of crucial proteins within the NF-κB pathway was examined via immunofluorescence and western blotting. The expression levels of downstream inflammatory factors, namely IL-1β and TNF-α, were detected through PCR. The effects of XJ-2 on the expression of skin barrier-related factors filaggrin (FLG), aquaporin 3 (AQP-3), and claudin1 (CLDN1) were investigated using PCR, immunofluorescence, and western blotting. Based on these findings, we comprehensively examined the mechanisms underlying the anti-inflammatory and barrier repair effects of XJ-2. XJ-2 primarily protected the internal structure and function of the cells by inhibiting the mass production of ROS and Ca^2+^ inflow. XJ-2 exerts anti-inflammatory effects by regulating the key proteins of the NF-κB/IKKα pathway and reducing the expression of inflammatory factors. XJ-2 repairs skin barrier damage by regulating multiple factors. Compound XJ-2 from *D. draco* exerts excellent anti-inflammatory and barrier repair effects, possesses great potential for the treatment of skin diseases, and can be used as a dermatological drug to repair skin barrier damage.

**Supplementary Information:**

The online version contains supplementary material available at 10.1038/s41598-025-01289-4.

## Introduction

*Daemonorops draco* Bl. is a plant belonging to *Daemonorops* (Arecaceae) in the palm family. The fruit secretes a reddish-brown resin (*Sanguis draconis*), which can be used as a dye in Chinese medicine. *D. draco* is the only source of Sanguis draconis listed in the Pharmacopoeia of the People’s Republic of China. *D. draco* possesses a round square or square brick shape with a dark red, shiny, hard, and brittle texture. The ground powder is brick-red with a slight gas and light taste^1^. *D. draco* promotes blood circulation, relieves pain, removes blood stasis, stops bleeding, generates muscle, and heals sores. The main chemical components of *D. draco* include flavonoids, diterpenoids, and other active compounds. Dracorubin and Dracorhodin are the main active ingredients of *D. draco*^2,3^. *D. draco* also possesses excellent pharmacological properties, including anti-inflammatory, antibacterial^4,5^, antioxidant^6^, antitumor^7,8^, wound healing^9,10^, and angiogenesis activities^11^. *D. draco* has great potential for the treatment of skin diseases and is widely used in skin health and other fields.

The skin barrier is the first line of defense in the human body. It protects the body from damage by irritating substances such as ultraviolet rays, prevents the loss of nutrients such as water and inorganic salts, and maintains homeostasis in the internal environment^12^. The physical barrier structure of the stratum corneum is the basis of the skin barrier function. It is primarily composed of keratinocytes and their intercellular fillers (such as lipids, natural moisturizing factors, and other components) that together form the unique “brick wall” structure of the skin barrier and constitute the first line of defense of the skin^13^. Skin hydration is an important mechanism that supports skin barrier function and is maintained by many highly hygrometric compounds in natural moisturizing factors (NMF). Filaggrin (FLG) is an important source of NMF that is conducive to maintaining normal cuticle hydration function^14,15^. Aquaporins are transporters of the cell membrane, and aquaporin 3 (AQP-3) is the main AQP in the skin. AQP-3 plays an important role in maintaining the skin barrier by affecting keratinocyte differentiation, proliferation, skin hydration, and other functions^16^. Tight junctions in the granular layer seal keratinocytes and form a second line of skin defense^17^. Claudins are the most important family of proteins that make up the tight junction structure. Claudins play an important role in the integrity of tight junctions and the function of the epidermal osmotic barrier. Knocking out claudin 1 (CLDN1) can cause severe skin barrier damage in mice^18^.

External environmental factors have a significant impact on the skin barrier, and light is one of the most important external factors responsible for damaging the skin barrier. Ultraviolet light and visible light can cause skin barrier damage, photoaging, and cancer. Ultraviolet light can induce pathological changes, such as oxidative stress, inflammation, and apoptosis of keratinocytes, and can destroy the structure and function of the skin barrier. The resistance of the skin to external stimuli is weakened, resulting in a massive loss of water and dry, rough, stinging, and itchy skin^19^.

Skin barrier damage caused by ultraviolet radiation is primarily caused by inflammatory reactions and oxidative stress. UVB irradiation induces the activation of TRPV4 ion receptor channels in cells, causing Ca^2+^ influx and disruption of intracellular Ca^2+^ homeostasis. High levels of Ca^2+^ stimulate the respiratory chain to produce more ROS, and the excessive accumulation of ROS can further target TRPV4 receptor channels and release more Ca^2+^^20^, ultimately resulting in a large accumulation of ROS and a cellular oxidative stress response. A large accumulation of ROS will further activate the NF-κB pathway, a heterodimer associated with inflammation that is composed of p50 and p65 subunits. ROS activates IKKα/IKKβ, phosphorylates and degrades IκB, releases NF-κB into the nucleus, activates the expression of cytokines related to immune inflammation such as IL1β, TNF-α, PGE-2, and others, releases inflammatory mediators, and induces skin inflammation. The inflammatory response, in turn, intensifies the accumulation of ROS, ultimately resulting in a vicious cycle and damage to the skin barrier^21^. The body’s inflammatory response triggers cellular changes and an immune response, which triggers a repair response in damaged tissue and cell growth at the inflammation site. If the cause of inflammation persists, the condition becomes chronic, leading to the failure of physiological control processes. In chronic inflammatory diseases, cellular alterations and growth can create a microenvironment that actively induces cancer development^22–24^.

The anti-inflammatory effects of *D. draco* extract have been reported, and *D. draco* extract has broad application prospects in skin health and medicine. However, the mechanism underlying the anti-inflammatory repair by *D. draco* compounds has not been reported. In this study, XJ-2, a compound with excellent anti-inflammatory repair activity in *D. draco*, was obtained using extraction and separation methods, and the mechanism of its anti-inflammatory and repair barrier activity was explored at the cellular level. These results provide a scientific foundation for the application of *D. draco* in medicine and skin health research.

## Results

### Seven compounds were obtained from *D. draco* by extraction and separation methods (XJ-1 ~ XJ-7)

**Fig. 1:**
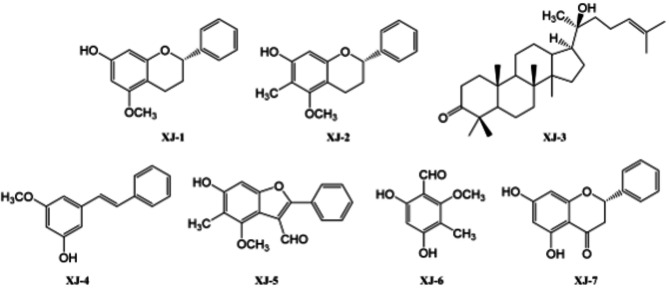
Chemical structures of seven compounds obtained by *D. draco* extraction and isolation.

After *D. draco* was crushed, petroleum ether, ethyl acetate, and 95% ethanol were refluxed with 5× petroleum ether, ethyl acetate, and 95% ethanol to obtain petroleum ether, ethyl acetate, and 95% ethanol extracts, respectively. The ethyl acetate fractions were extracted using silica gel column chromatography to obtain 22 flow fractions (Fr.A ~ Fr.V). Seven compounds were obtained by silica gel column chromatography, ODS column chromatography, SephadexLH-20 gel column chromatography, recrystallization, and other extraction and separation methods. Their chemical structures are shown in Fig. 1.

### Establishment of a UVB-mediated HaCaT cell damage model

**Fig. 2:**
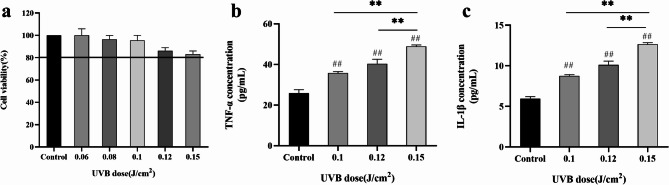
Effect of different UVB irradiation doses on cell survival rate and secretion of TNF-α and IL-1β. ^#^*P* < 0.05, ^##^*P* < 0.01. TNF-α, tumor necrosis factor-α; IL-1β, interleukin-1β; UVB, ultraviolet B.

Cell Counting Kit-8 (CCK-8) assay was used to determine the effects of different doses of UVB irradiation on the viability of HaCaT cells (Fig. 2a). When the UVB irradiation doses were 0.06, 0.08, 0.1, 0.12, and 0.15 J/cm^2^, the survival rate of the cells was greater than 80%. The cell survival rate decreased with increasing UVB doses. Therefore, a UVB dose with a cell survival rate > 80% was selected as the safe dose.

TNF-α and IL-1β are significant inflammatory markers in HaCaT cells, and the overexpression of TNF-α and IL-1β can cause cell inflammation, differentiation, and apoptosis that are associated with a variety of diseases^25–29^. In this study, compared to the blank Control group, the contents of TNF-α and IL-1β secreted by the cells in the UVB irradiation dose group of 0.1–0.15 J/cm^2^ were significantly increased (*P* < 0.01; Fig. 2b and c). In the 0.15 J/cm^2^ UVB irradiation group, the levels of TNF-α and IL-1β were 48.88 pg/mL and 12.66 pg/mL, respectively. UVB irradiation significantly induced the secretion of the inflammatory cytokines TNF-α and IL-1β. Therefore, the optimal irradiation dose for establishing a UVB-induced inflammatory model was 0.15 J/cm^2^.

### Effects of XJ-1 ~ XJ-7 on the viability of HaCaT cells

**Fig. 3:**
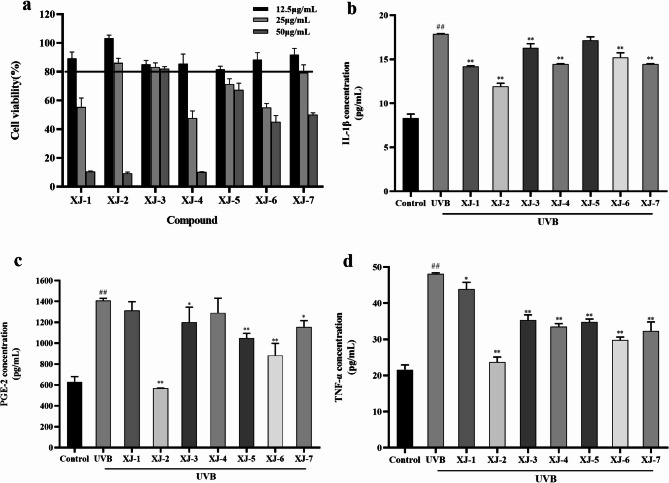
Effect of XJ-1 ~ XJ-7 on the survival rate of HaCaT cells, and the expression of inflammatory factors in UVB-induced HaCaT cells. ^#^*P* < 0.05, ^##^*P* < 0.01, compared to blank; ^*^P < 0.05, ^**^P < 0.01, compared to UVB.

The effects of XJ-1 ~ XJ-7 on HaCaT cell activity were also determined. As shown in Fig. 3a, the cell survival rates decreased with increasing concentrations of the seven samples. At 25 µg/mL, XJ-2 and XJ-3 exhibited no toxicity to HaCaT cells. At 12.5 µg/mL, XJ-1, XJ-4, XJ-5, XJ-6, and XJ-7 exhibited no toxic effects on HaCaT cells. Therefore, in the subsequent experiment, the concentration of XJ-1 ~ XJ-7 was selected to be 12.5 µg/mL for comparison of uniform sample concentration.

### Effects of XJ-1 ~ XJ-7 on inflammatory markers in UVB-mediated HaCaT cells

The expression of the inflammatory factors IL-1β, TNF-α, and PGE-2 in HaCaT cells was detected by enzyme-linked immunosorbent assay (ELISA). Our results demonstrated (Fig. 3b, c and d) that the expression of IL-1β, TNF-α, and PGE-2 in HaCaT cells irradiated by UVB was significantly increased (*P* < 0.01). The expression of IL-1β, TNF-α, and PGE-2 in HaCaT cells decreased after treatment with 12.5 µg/mL of XJ-1 to XJ-7, and the expression of PGE-2, TNF-α, and IL-1β in HaCaT cells was significantly inhibited by XJ-2 (*P* < 0.01). Thus, XJ-2 significantly inhibited UVB-induced inflammatory response in HaCaT cells. Future experiments should consider XJ-2 as the main research object to explore the mechanisms underlying its anti-inflammatory and repair functions.

### Effects of XJ-2 on the expression of CLDN1, AQP-3, and FLG in the skin with UVB-mediated barrier damage

**Fig. 4:**
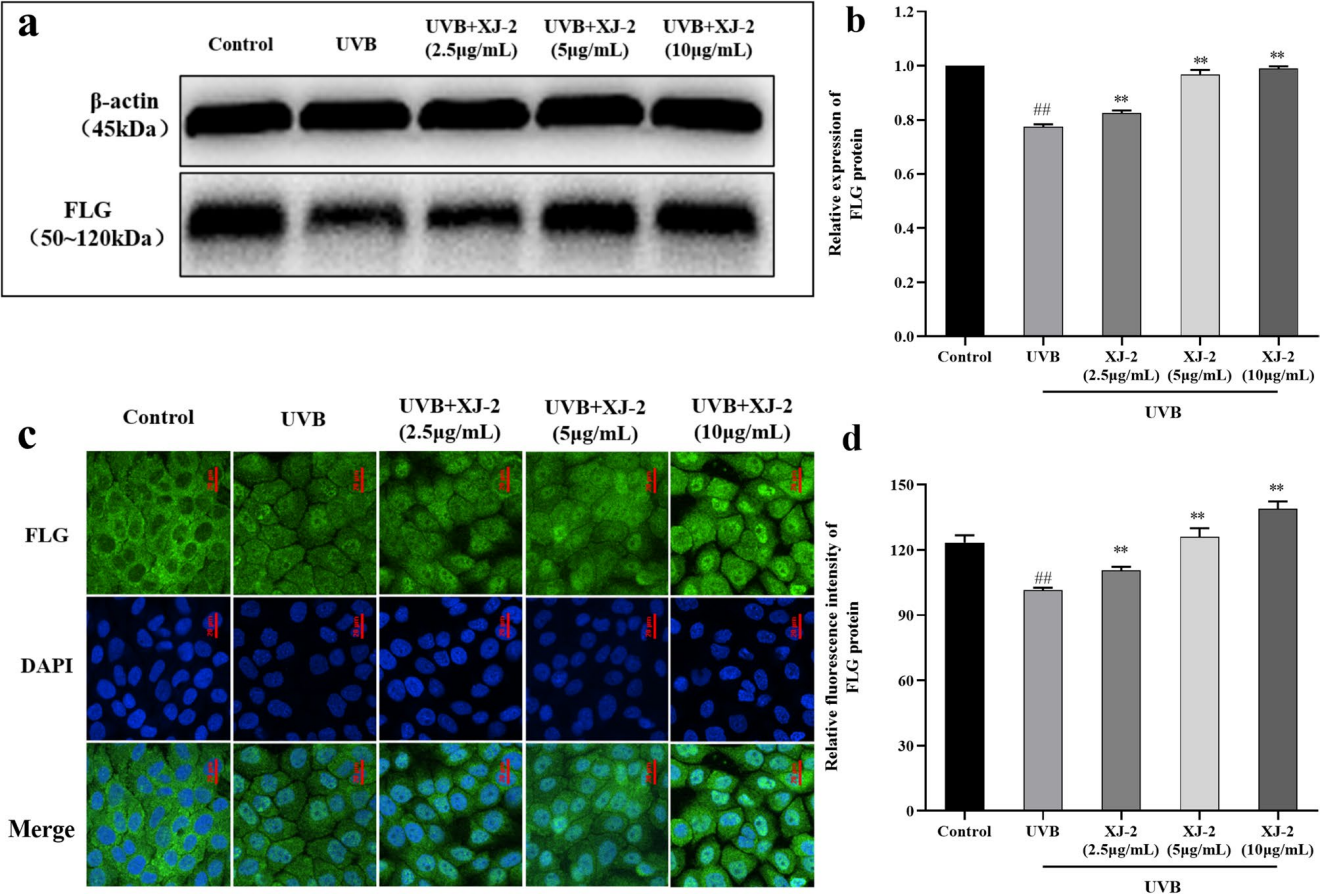
Western blotting and immunofluorescence showing the FLG levels in HaCaT. ^##^*P* < 0.01 compared to the Control; ^*^P < 0.05/^**^P < 0.01, compared to the UVB.

FLG is the skeleton of the stratum corneum and is connected to keratin fibers for regular aggregation. Simultaneously, FLG can be hydrolyzed to produce a NMF under the action of enzymes that maintain the hydration function of the stratum corneum, which is indispensable for skin barrier function^17^. Western blotting, immunofluorescence, and PCR revealed that XJ-2 significantly increased FLG expression after HaCaT cell injury and effectively repaired the skin barrier (Figs. 4 and 7a).

**Fig. 5:**
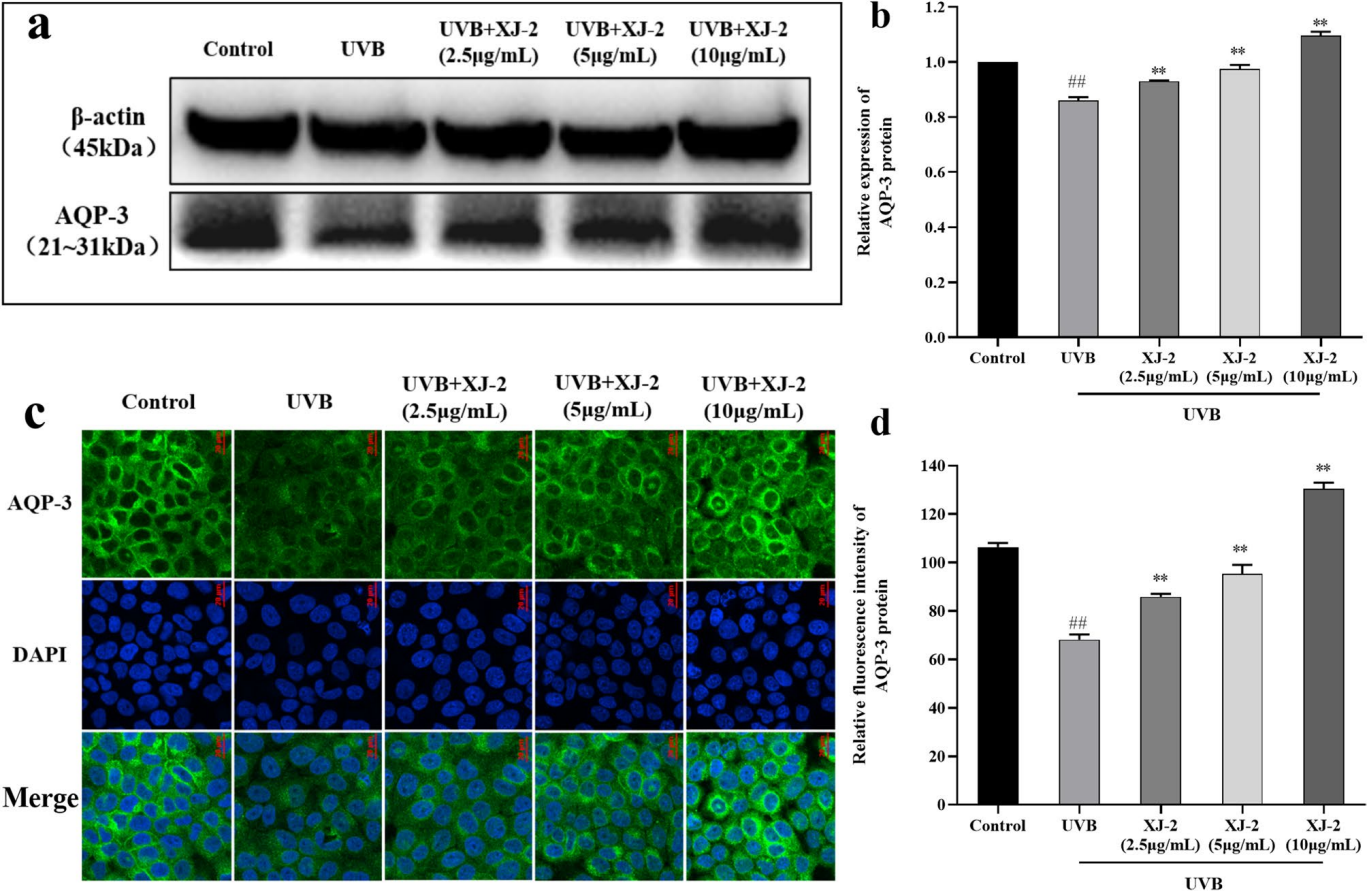
Western blotting and immunofluorescence showing the AQP-3 levels in HaCaT. ^##^*P* < 0.01 compared to the Control; ^*^P < 0.05/^**^P < 0.01, compared to the UVB.

**Fig. 6:**
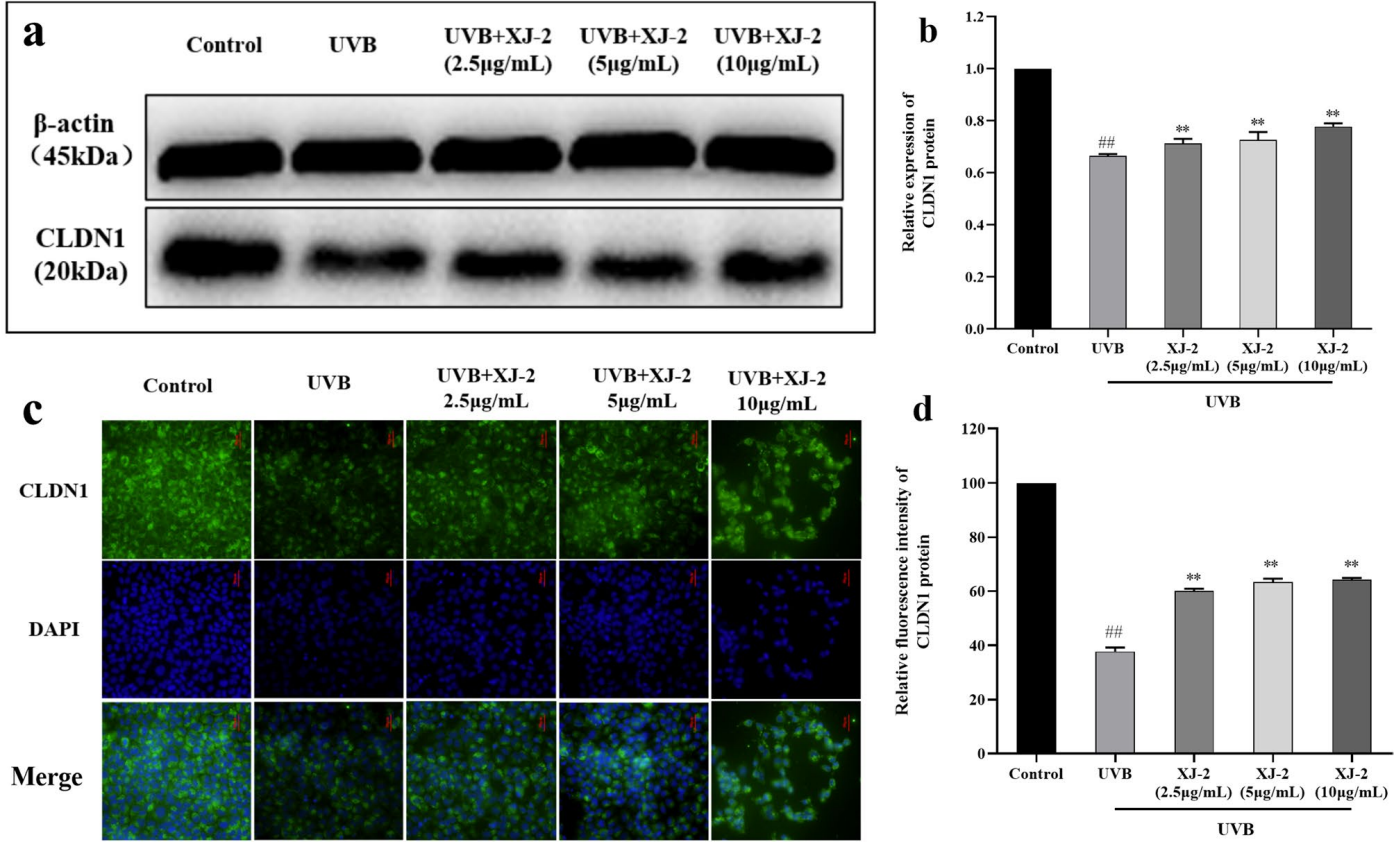
Western blotting and immunofluorescence showing the CLDN1 levels in HaCaT. ^##^*P* < 0.01 compared to the Control; ^*^P < 0.05/^**^P < 0.01, compared to the UVB.

**Fig. 7:**
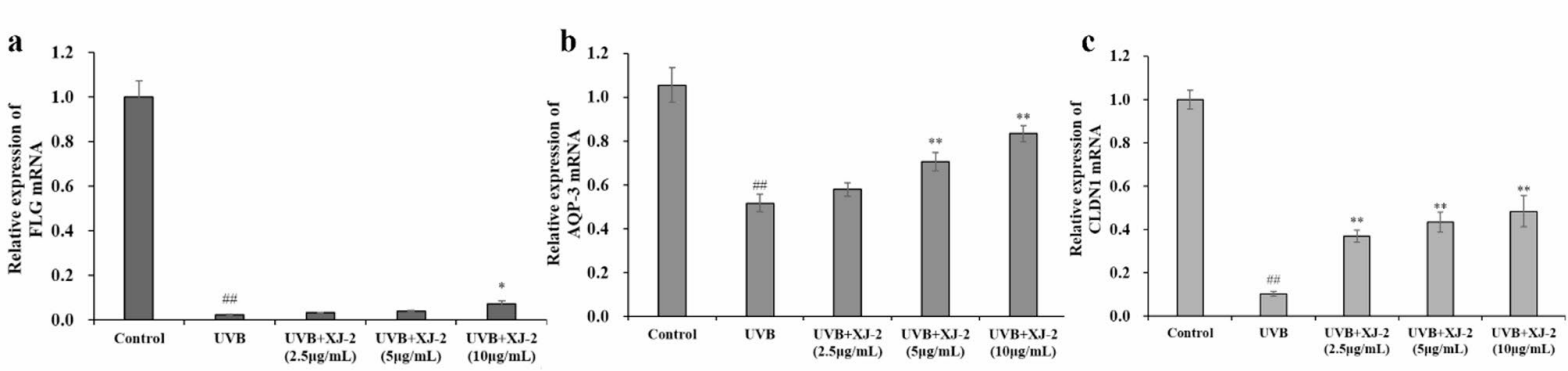
PCR showing the FLG, AQP-3, and CLDN1 levels in HaCaT. ^##^*P* < 0.01 compared to the Control; ^*^P < 0.05/^**^P < 0.01, compared to the UVB.

AQP-3 is a transmembrane transporter involved in the permeability of water molecules in keratinocytes and the regulation of water and ion levels inside and outside the cell, and is a key factor in maintaining skin hydration^30^. CLDN1 plays an important role in tight junction integrity and epidermal osmotic barrier function^31^. Western blotting, immunofluorescence, and PCR results indicated that XJ-2 significantly increased the gene and protein expression of AQP-3 after HaCaT cell injury (Figs. 5 and 7b). Immunofluorescence, PCR, and western blotting analyses demonstrated that XJ-2 significantly increased the gene and protein expression of CLDN1 after HaCaT cell injury and maintained epidermal tight junctions and skin hydration (Figs. 6 and 7c). This protects the integrity of the skin barrier.

### Effects of XJ-2 on ROS expression in UVB-mediated HaCaT cells

**Fig. 8:**
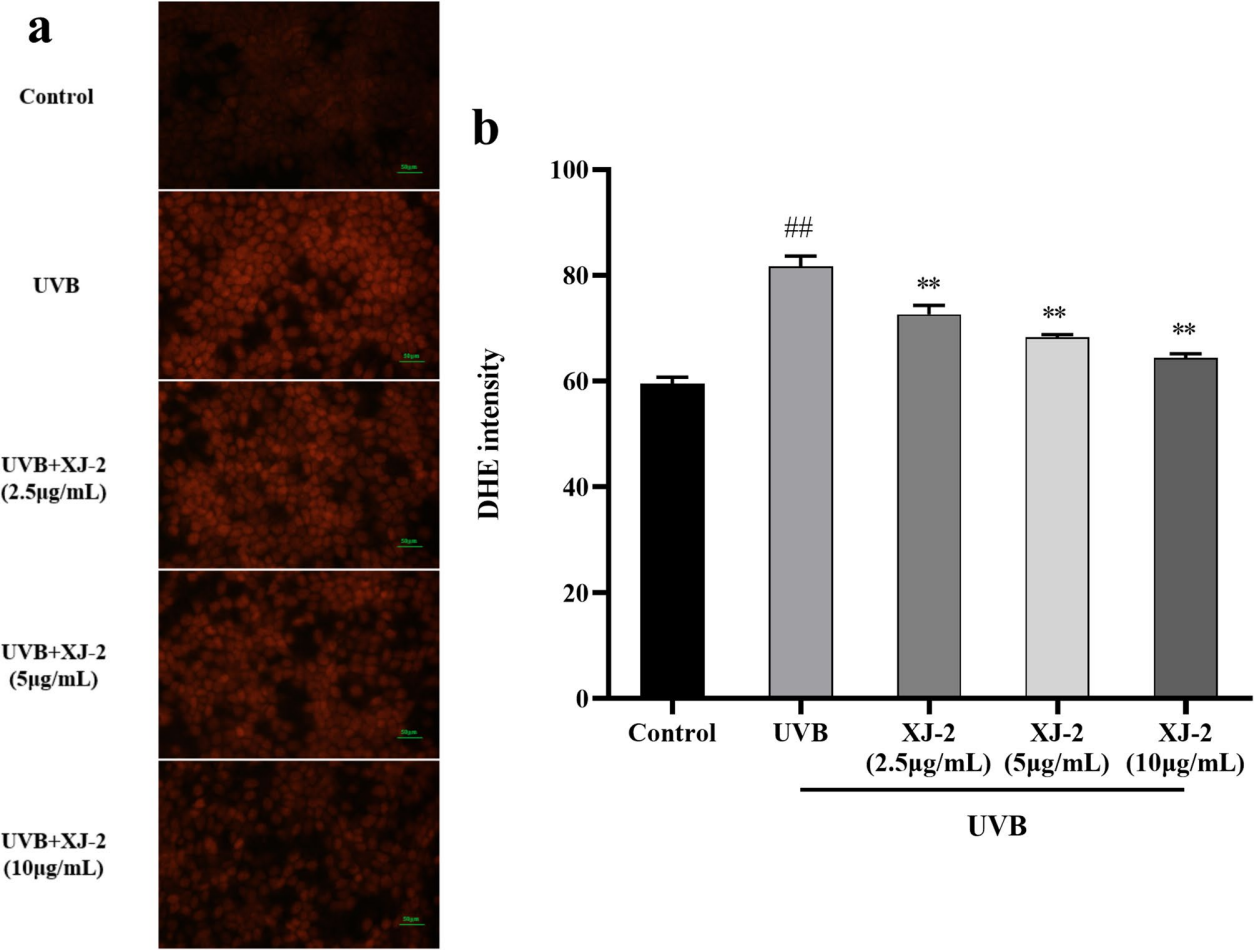
XJ-2 prevents intracellular ROS generation in HaCaT cells as observed with DHE staining. DHE, dihydroethidium.

In the current study, ROS levels significantly increased in UVB-irradiated HaCaT cells. Our results revealed (Fig. 8) that dihydroethidium (DHE) fluorescence was significantly increased in UVB-irradiated HaCaT cells owing to ROS production (*P* < 0.01). After XJ-2 treatment, the fluorescence intensity of DHE decreased significantly in a dose-dependent manner (*P* < 0.01). Therefore, XJ-2 treatment significantly inhibited intracellular ROS production, reduced intracellular oxidative stress, and protected cell structural integrity.

### Regulation of Ca^2+^ in UVB-irradiated HaCaT cells by XJ-2

**Fig. 9:**
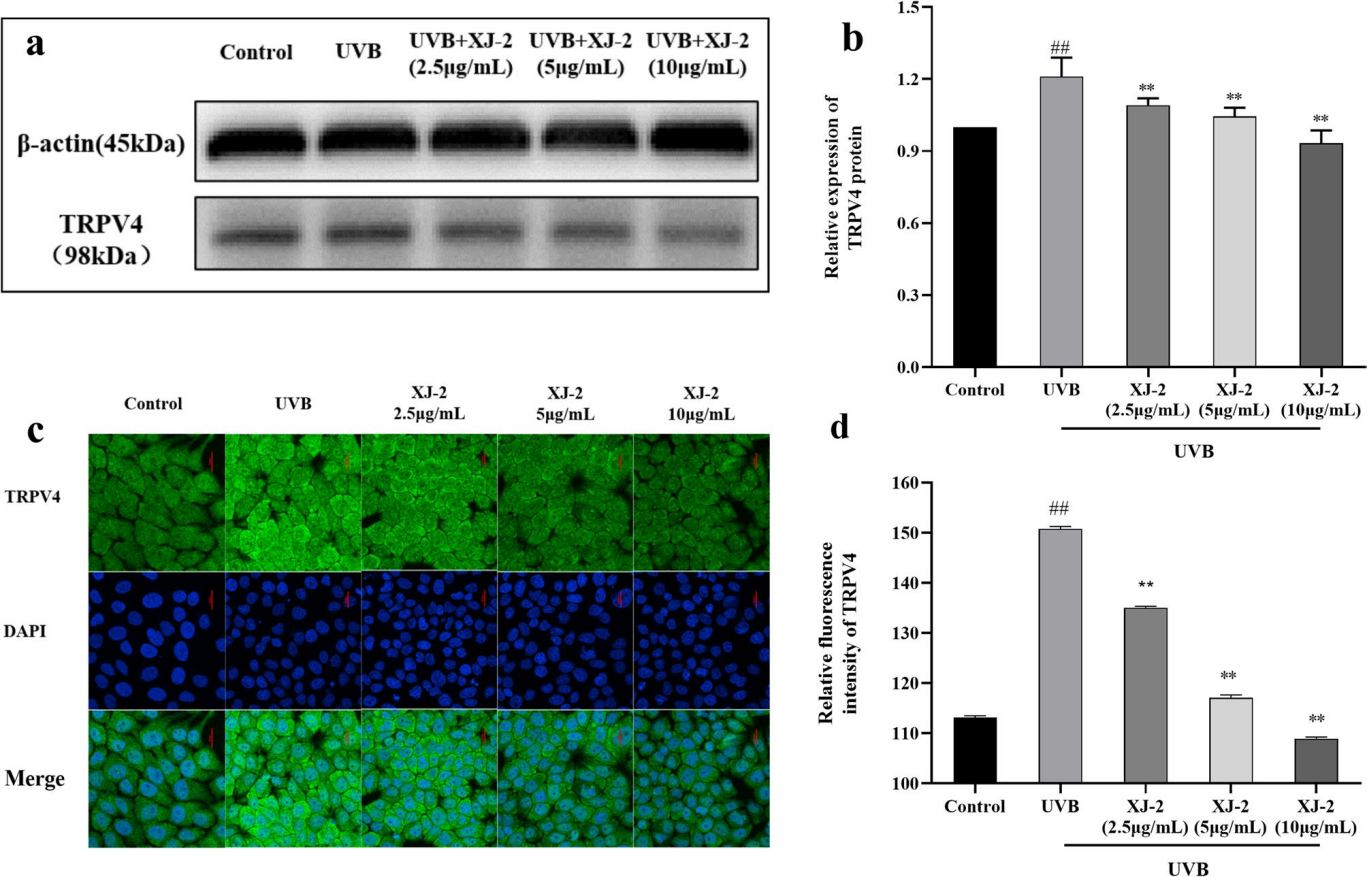
Western blotting and immunofluorescence showing the TRPV4 levels in HaCaT. ^##^*P* < 0.01 compared to the Control; ^*^P < 0.05/^**^P < 0.01, compared to the UVB.

**Fig. 10:**
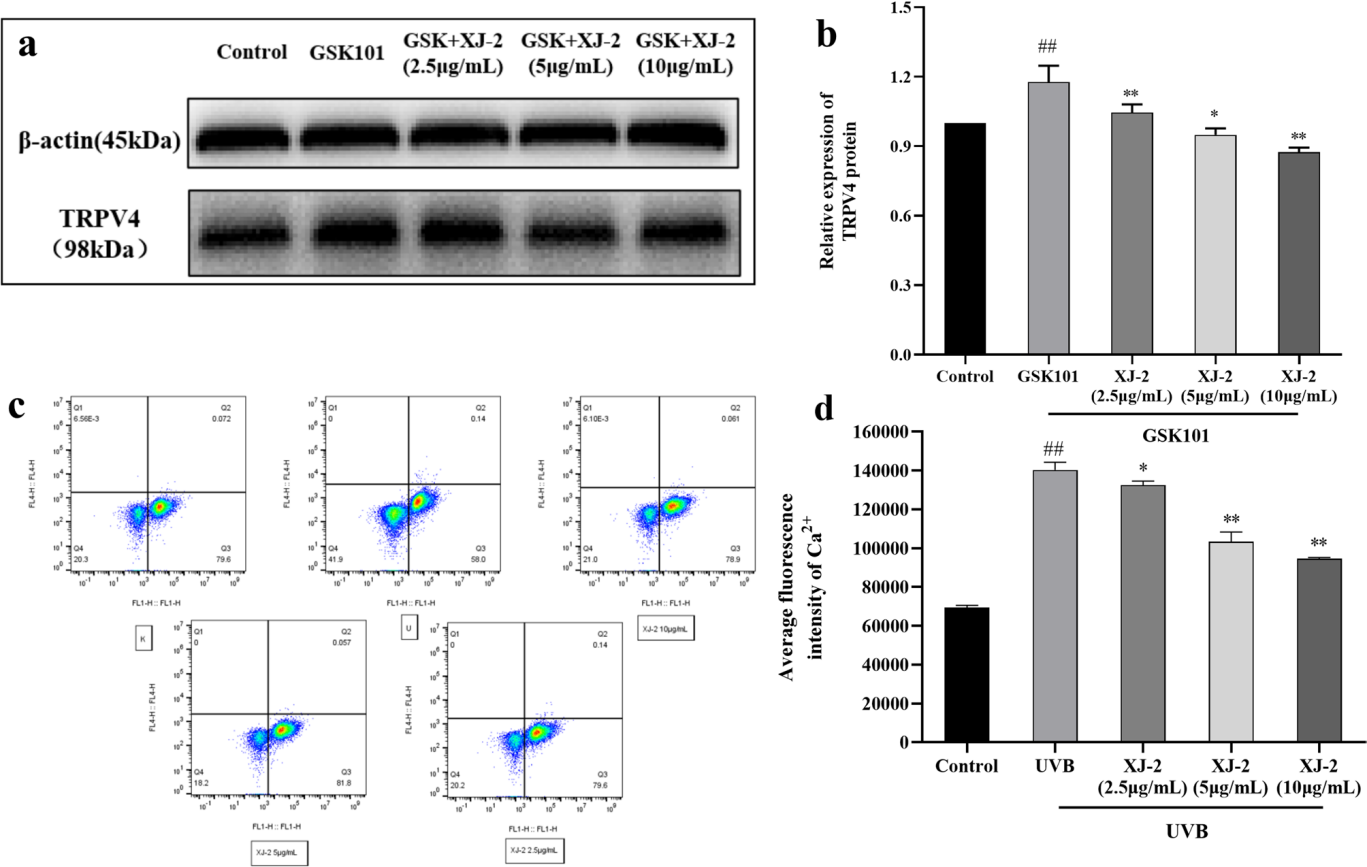
Western blotting showing the TRPV4 levels in GSK101 treated HaCaT. Flow cytometry indicating the Ca^2+^ levels in HaCaT cells. ^##^*P* < 0.01 compared to the Control; ^*^P < 0.05, compared to the UVB, ^**^P < 0.01, compared to the UVB.

TRPV4 is a nonselective Ca^2+^ channel protein that mediates Ca^2+^ influx after activation and is involved in the regulation of various physiological functions^32^. Immunofluorescence and western blotting detection of the expression of TRPV4 receptor protein on HaCaT cells demonstrated that UVB irradiation activated the expression of TRPV4 protein (Fig. 9), and the activation of TRPV4 led to the increase in intracellular Ca^2+^ concentration and the release of chemokines, ultimately causing inflammation and additional damage. The fluorescence intensity of TRPV4 protein in UVB cells treated with XJ-2 significantly decreased in a dose-dependent manner (*P* < 0.01). In addition, HaCaT cells were treated with TRPV4 agonists. In XJ-2 treated GSK101^33^ agonist cells, the expression of TRPV4 protein significantly decreased in a dose-dependent manner (*P* < 0.01) (Fig. 10a and b). Thus, XJ-2 significantly reduced the expression level of TRPV4 in UVB-damaged cells, thereby preventing skin damage caused by Ca^2+^ influx.

Flow cytometry detected the level of Ca^2+^ in the cells and indicated that, compared to the blank Control group, the level of Ca^2+^ in the UVB irradiation group increased sharply (*P* < 0.01). As a secondary messenger of Ca^2+^, intracellular Ca^2+^ overload produces several free radicals that increase cell membrane permeability and cause a large influx of Ca^2+^, ultimately resulting in a vicious cycle. Eventually, cell function is damaged and death can occur^34^. The Ca^2+^ concentration in cells treated with XJ-2 decreased in a dose-dependent manner (Fig. 10c and d). Therefore, XJ-2 significantly reduced the level of Ca^2+^ in injured cells, effectively protecting their internal function and preventing skin damage caused by Ca^2+^ influx.

### Effects of XJ-2 on the IKKα /NF-κB p65 signaling cascade in HaCaT cells with UVB-mediated injury

NF-κB is a major factor that mediates the UVB-induced inflammatory response by inducing a variety of pro-inflammatory proteins^35^.

**Fig. 11:**
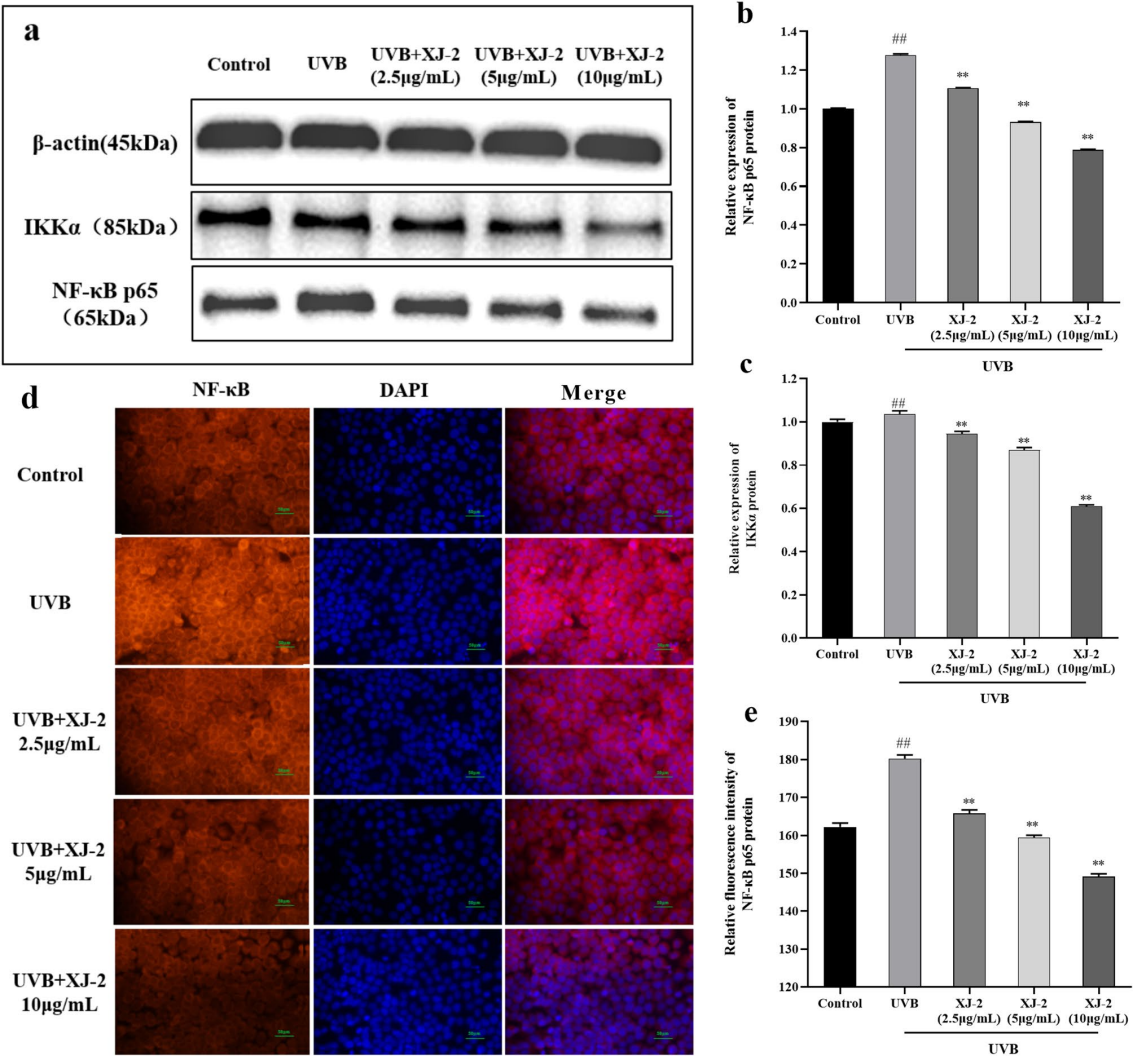
Western blotting showing the IKKα and NF-κB p65 levels in HaCaT cells (a, b, and c). Immunofluorescence showing the NF-κB p65 levels in HaCaT cells (d, e). ^##^*P* < 0.01 compared to the Control; ^**^P < 0.01, compared to the UVB.

**Fig. 12:**
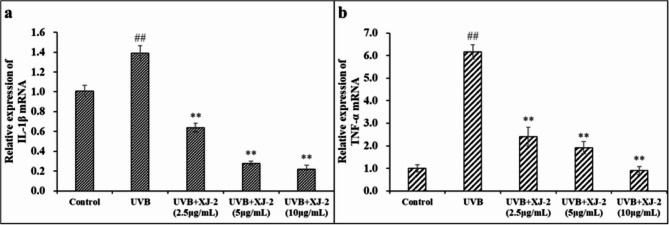
RT-PCR showing the IL-1β (a) and TNF-α (b) levels in HaCaT cells. ^##^*P* < 0.01 compared to the Control; ^**^P < 0.01, compared to the UVB.

The key components of the NF-κB signaling pathway were detected by western blotting and immunofluorescence. The results showed that under UVB stimulation, the IKKα/NF-κB signal was activated in HaCaT cells. Specifically, the p65 subunit protein of NF-κB was activated, and active nuclear translocation occurred. Moreover, the activation of the NF-κB pathway upregulated pro-inflammatory cytokines, namely IL-1β and TNF-α. These pro-inflammatory cytokines activate inflammatory signaling pathways and induce inflammatory responses. Treatment with XJ-2 significantly inhibited the expression of the IKKα/NF-κB protein and the translocation of the NF-κB p65 subunit to the nucleus induced by UVB (Fig. 11), thereby downregulating the expression of IL-1β and TNF-α, which are pro-inflammatory cytokines (Fig. 12), and effectively reducing the inflammatory damage of cells. Therefore, XJ-2 effectively reduced inflammatory damage caused by UVB radiation in HaCaT cells by regulating the NF-κB signaling pathway.

## Discussion

As a traditional Chinese medicine, *D. draco* has potential applications in the repair of skin damage; however, its mechanism of action remains unclear. In this study, we obtained monomers XJ-1–XJ-7 from *D. draco* using extraction and separation technologies. We obtained seven monomers (XJ-1–XJ-7) using extraction and separation technology, and simulated skin barrier damage caused by ultraviolet light by UVB induction in HaCaT cells. It was observed that XJ-1 ~ XJ-7 inhibited the expression of inflammatory factors IL-1β, TNF-α, and PGE-2. XJ-2 exerted significant anti-inflammatory effects. Further investigation into the repair mechanism of XJ-2 in skin barrier injury demonstrated that XJ-2 plays an anti-inflammatory role by inhibiting oxidative stress and Ca^2+^ influx in cells, thereby inhibiting the expression of key proteins of the NF-κB inflammatory pathway and enhancing the expression of repair and moisturizing factors in injured cells.

The skin barrier functions primarily through the stratum corneum of the epidermis and is typically composed of keratinocytes, intercellular lipids, and keratinocyte desmosomes. FLG is involved in the formation of a stable keratinoid envelope called the cornified envelope (CE), which promotes epidermal differentiation. In the stratum corneum, FLG is the main barrier against physical stimulation by the external environment, and it hydrolyzes to form a NMF, which acts as a hydration and water-retention factor^36,37^. AQP-3 is an aquaglyceroporin found on the skin cell membrane, which is a channel specifically used for transporting water, controlling the entry and exit of water and nutrients in skin cells, and regulating intracellular water to maintain the epidermal water permeability barrier^38^. The tight junction structure of the side walls of keratinocytes in the granular layer of the epidermis is an important part of the physical barrier function of the skin, and CLDN1 plays an important role in the integrity of tight junctions and the function of the epidermal osmotic barrier^39^. Therefore, FLG, AQP-3, and CLDN1 are all closely related to skin barrier function, and the absence of these factors can cause serious damage to the skin barrier. Our results revealed that XJ-2 significantly increased the expression of FLG, AQP-3, and CLDN1 in cells after UVB damage, indicating that XJ-2 can repair the damaged skin barrier, maintain tight skin connections and skin hydration, and maintain homeostasis of skin barrier structure and function.

After exposure to UVB, the mitochondrial function of HaCaT cells was damaged, ultimately causing ROS generation and oxidative DNA damage^40^. Excessive ROS accumulation can further target the TRPV4 ion receptor channels, and TRPV4 in HaCaT cells is directly involved in the development of pruritus. The activation of TRPV4 channels in keratinocytes can cause scratching behavior, induce Ca^2+^ inflow, and induce itch signal transmission from the skin to the peripheral nerve and then to the center, ultimately resulting in immune inflammation and damage to the skin barrier^41^. Our results showed that XJ-2 significantly inhibited ROS accumulation, reduced the expression level of TRPV4, and regulated Ca^2+^ homeostasis. Therefore, we inferred that XJ-2 may play a role in relieving itching by reducing ROS levels and decreasing the expression of TRPV4 to reduce intracellular oxidative stress levels.

The massive accumulation of ROS induced by UVB activates the NF-κB pathway, a heterodimer associated with inflammation composed of p50 and p65 subunits. ROS activates IKKα/IKKβ, and this releases NF-κB into the nucleus and activates the expression of immunoinflammatory-related factors such as IL-1β, TNF-α, PGE-2, and others^42^. TNF-α is a pro-inflammatory factor widely distributed in the body that can mediate a variety of physiological and biochemical reactions such as inflammation, apoptosis, immune response, and others^43^. IL-1β is a key pro-inflammatory cytokine involved in a variety of autoimmune inflammatory responses and a variety of cellular activities, including cell proliferation, differentiation, and apoptosis. As an important pro-inflammatory mediator, PGE-2 is involved in inflammation and can cause edema, redness, pain, and other symptoms^44^. Our study illustrated that XJ-2 can significantly inhibit the activity of IKKα in cells after UVB injury and then inhibit the activation of NF-κB and the migration of p65 into the nucleus, reducing the expression of inflammatory factors IL-1β, TNF-α, and PGE-2, and reduce the occurrence of inflammatory response. Therefore, we inferred that XJ-2 could mitigate UVB-induced inflammation by inhibiting the NF-κB signaling pathway. Nevertheless, further investigations of the signaling pathways and animal experiments are required to confirm the reliability of XJ-2 in alleviating itching and skin inflammation.

As a rare medicinal herb, *D. draco*. exhibits remarkable pharmacological activities. However, research on its active ingredients and mechanisms of action is scarce, leaving a gap in the theoretical basis.

In this study, seven compounds were successfully extracted from D. draco. Among these, XJ-2 has a unique biological activity. It can inhibit the generation of ROS and the inward flow of calcium ions, thus playing a cytoprotective role. XJ-2 can reduce the release of inflammatory factors by modulating the NF-κB/IKKα signaling pathway to achieve an anti-inflammatory effect and regulate skin barrier-related proteins to promote skin barrier repair. It has great potential for the development of dermatological therapeutic drugs and skin care products, especially for the treatment of inflammatory dermatological diseases, such as atopic dermatitis and psoriasis, and is expected to become a novel drug or efficient skincare ingredient.

However, that study was limited to the cellular level. The molecular mechanisms and exact targets remain to be elucidated, and there is a lack of animal experiments or 3D skin models for validation. Therefore, future studies should use animal models or 3D skin to verify the skin barrier repair effects and safety of XJ-2. Moreover, modern molecular biology techniques should be used to analyze their targets and signaling pathways and molecular docking technology should be employed to optimize the structure of the compound and enhance its biological activity. These efforts will fill research gaps and enhance the credibility of the conclusions. This will not only help promote progress in the field of dermatological treatment but also provide new ideas for the in-depth development and utilization of herbal medicines, demonstrating the broad prospects and far-reaching significance of herbal medicines in modern medical research.

## Conclusions

**Fig. 13:**
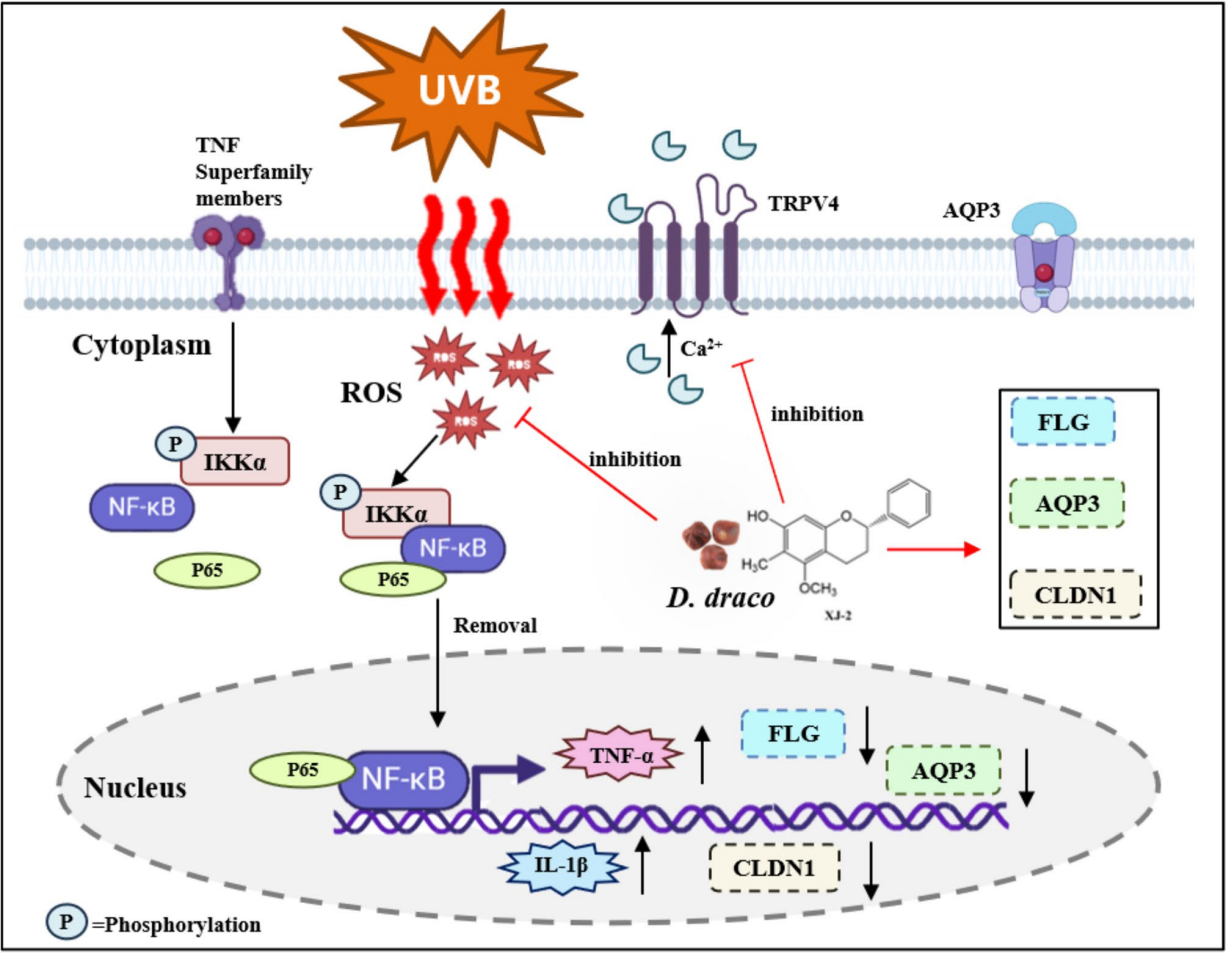
Mechanism of XJ-2 in repairing UVB-induced skin damage: inhibiting the NF-κB pathway to alleviate inflammation and modulating barrier protein expression to restore the skin barrier.

In traditional Chinese medicine, *D. draco* exerts various beneficial effects. Its composition is complex and it possesses a unique structure^45^. *D. draco* extract has been widely used in dermatology and medicine. Therefore, it is necessary to study its composition, biological activity, and mechanism of action to broaden our understanding of the structure-activity relationship of *D. draco* components. In this study, the active monomer, XJ-2, from *D. draco*, which has excellent anti-inflammatory activity, was obtained using extraction and separation methods. XJ-2 also effectively increased the expression of FLG, AQP-3, and CLDN1 in cells after UVB injury and promoted the repair of the damaged skin barrier. We observed that XJ-2 inhibited the production of excess ROS and Ca^2+^ inflow in cells and reduced the oxidative damage, thus inhibiting the activation of the NF-κB inflammatory pathway, ultimately reducing the expression of inflammatory factors such as TNF-α and IL-1β in cells and attenuating the inflammatory response (Fig. 13). Therefore, we propose that XJ-2 is an effective anti-inflammatory and barrier repair agent. Our study provides a scientific basis for the use of *D. draco* in skin healthcare.

## Materials and methods

### Reagents and instruments

HaCaT cells were purchased from the Meisen Chinese Tissue Culture Collection (Meisen CTCC, Zhejiang, China). Dulbecco’s modified Eagle’s medium (DMEM), penicillin–streptomycin (PS), heat-inactivated fetal bovine serum (FBS), and trypsin-EDTA solution A (0.25% trypsin and 0.02% EDTA) were purchased from Gibco ( Waltham, MA, USA). Cell Counting Kit-8, Fluo-4 Calcium Assay Kit, and ROS fluorescent probe DHE were obtained from Biorigin (Beijing, China). Human PGE-2 ELISA kit, Human TNF-α ELISA kit, and Human IL-1β ELISA kit were obtained from Biotechwell (Shanghai, China). The antibodies used for western blotting analysis were acquired from Abcam (Shanghai, China) and Cell Signaling Technology (Danvers, MA, USA). GSK101 was purchased from AmBeed. UVJLY-I was acquired from Beijing Evenpure Scientific Instrument Technology (Beijing, China).

The *D. draco* used in this study (production lot number: 1808549131, origin: Malaysia) was purchased from Anguo Qi’ao Traditional Chinese Medicine Drinking Tablets, Baoding, Hebei Province, China.

### Extraction of *D. draco*

After *D. draco* was crushed, 5× petroleum ether, ethyl acetate, and 95% ethanol were used to extract the petroleum ether, ethyl acetate, and 95% ethanol. A total of 22 flow fractions (fractions A–V) were obtained using silica gel column chromatography. Fr. H. was separated and purified by silica gel column chromatography, ODS column chromatography, and SephadexLH-20 gel column chromatography and then recrystallized to obtain XJ-1, XJ-3, XJ-4, XJ-5, XJ-6, and XJ-7. Fr.F was separated by silica gel column chromatography and recrystallized to obtain compound XJ-2.

### Cell culture

HaCaT cells were cultured in DMEM culture medium containing 10% FBS and 1% PS at 37 °C and 5% CO_2_ until cell fusion reached 80–90%, followed by passaging. The medium was removed and the cultures were rinsed twice with PBS. Conventional digestion with 0.25% trypsin was performed for 8 min, followed by rapid termination of digestion with the medium. After centrifugation at 1,000 rpm for 5 min, remove the supernatant from the centrifuge tubes, and resuspend the cells in complete medium. The cell suspension was cultured in DMEM supplemented with 10% FBS and 1% PS. When the cultured cells reached approximately 80% confluence, they were ready for use in the experiments.

### UVB irradiation and drug treatment

HaCaT cells were inoculated in six-well plates at a density of 6 × 10^5^ cells/well. When HaCaT cells reached 80-90% fusion, the medium was removed and the cells were washed twice with PBS. Subsequently, the HaCaT cells were covered with PBS and irradiated with UV light (UVJLY-I, UV wavelength: 254, 312, and 365 nm, Beijing, China) at 0.06, 0.08, 0.1, 0.12 and 0.15 J/cm^2^. Immediately after irradiation, PBS was removed from the wells and appropriate amounts of serum-free medium or sample solution (serum-free medium preparation) were added and incubated for 24 h. The cell viability and the expression levels of TNF-α, and IL-1β were detected at different UVB irradiation doses to determine the optimal UVB irradiation dose. The cell viability and the expression levels of PGE-2, TNF-α, and IL-1β were detected at 0.15 J/cm^2^ UVB irradiation doses to determine the anti-inflammatory effects of XJ-1 to XJ-7.

### Cell viability assay

Cell viability was determined using the CCK-8. HaCaT cells were pretreated with different doses of UVB or different concentrations of sample solutions. After 24 h of incubation at 5% CO_2_ and 37 °C, 10 µL of CCK-8 solution was added to the cells in the dark. Subsequently, the reaction was carried out for 1 h at 37 °C, protected from light, and the cell viability was calculated by measuring the absorbance of the samples at 450 nm using a microplate reader (TECAN, Männedorf, Switzerland).

### Enzyme-linked immunosorbent assay

HaCaT cells were divided into blank Control, UVB irradiation, and experimental groups (the sample solution was added after UVB irradiation). HaCaT cells were seeded in six-well plates at a density of 6 × 10^5^ cells/well. When the cells reached 80-90% fusion, the medium was removed, and the cultures were rinsed twice with 1 mL of PBS. Subsequently, 1 mL PBS was added to each well to cover the cells before UVB irradiation. At the end of the irradiation, the wells were cleared with PBS, and an appropriate amount of serum-free medium or sample solution was added. The cultures were incubated at 37 °C and 5% CO_2_ for 24 h. The expression of PGE-2, TNF-α, and IL-1β was detected using ELISA kits.

### Cellular Ca^2+^ assay by flow cytometry

HaCaT cells and sample solutions were added as described previously. After 24 h of culture, HaCaT cells were digested with trypsin, resuspended in the culture medium, washed once with PBS, and centrifuged. Fluo-4 staining solution (1 mL) was added to the precipitate obtained from the previous centrifugation and resuspended the cells in the staining solution. The cells were then incubated at 37℃ for 30 min under light protection. After incubation, the cells were analyzed directly using flow cytometry at Ex/Em = 490/525 nm.

### Fluorescent probe detection of ROS expression in cells

HaCaT cells and sample solutions were added as described previously. After 24 h of incubation, the cell culture medium was removed and an appropriate amount of diluted DCFH-DA probe solution was added. DCFH-DA was diluted in serum-free medium at a ratio of 1:1000 to a final concentration of 10 µM. HaCaT cells were incubated for 30 min at 37 °C. The cells were washed three times with PBS to completely remove DCFH-DA that had not entered the cells. After washing, the cells were observed under a laser confocal microscope.

### Immunofluorescence assay

HaCaT cells and sample solutions were added as described previously. After 24 h of culture, HaCaT cells were fixed with 4% paraformaldehyde for 30 min at 4 °C and then infiltrated with PBS containing 0.2% TritonX-100 for 20 min. Cells were incubated with an antibody (1:200 in 5% BSA) overnight at 4 °C, then infiltrated with goat anti-mouse IgG Dylight 488 (1:500) for 2 h at room temperature (25 °C). After staining with DAPI for 10 min, the cells were visualized using an inverted fluorescence microscope.

### Quantitative real-time PCR

HaCaT cells and sample solutions were added as described previously. Total RNA was isolated from the cells using TRIzol reagent (Beyotime) after 24 h. RNA was quantified using a UV spectrophotometer and RNA quality was determined using the OD260/OD280 ratio. The corresponding cDNA was obtained by reverse transcription of the RNA, and the results were normalized to GAPDH expression and calculated using the ImageJ software.

### Western blotting analysis

HaCaT cells and sample solutions were added as described previously. After 24 h of culture, HaCaT cells were digested with trypsin, resuspended in the culture medium, and washed once with PBS. The cell precipitate was resuspended in 100 µL of cell lysis buffer (cell lysis buffer was prepared at a ratio of total protein lysate: phosphatase inhibitor: protease inhibitor: PMSF = 100:1:1:1). Total protein concentration was determined using the BCA reagent at 562 nm.

Equal amounts of proteins (30 µg) were electrophoresed on sodium dodecyl sulfate-polyacrylamide gel electrophoresis (SDS-PAGE), and then the separated protein bands were transferred to a polyvinylidene difluoride (PVDF) membrane. The membranes were incubated with primary antibodies overnight, developed with horseradish peroxidase (HRP)-conjugated secondary antibodies, and developed using an ECL ultrasensitive chemiluminescence kit. Samples were scanned using a gel imaging system (Tanon, Shanghai, China) and quantified using ImageJ software (National Institutes of Health, Bethesda, MD, USA).

### Statistical analysis

All data are expressed as mean ± standard deviation (SD) of three independent experiments. One-way analysis of variance (ANOVA) followed by Tukey’s test and *t*-test was performed using Prism software (GraphPad Software, La Jolla, CA, USA) to assess the statistical significance of the Control group.

## Electronic supplementary material

Below is the link to the electronic supplementary material.

Supplementary Material 1

## Data Availability

The datasets generated during and/or analysed during the current study are available from the corresponding author on reasonable request.
