# Supplementary figures and images for: Anti-inflammatory and barrier repair mechanisms of active components in Daemonorops draco Bl. for UVB-induced skin damage
Source: Sci Rep. 2025 May 17;15:17124. doi: 10.1038/s41598-025-01289-4 (PMC12085626; doi:10.1038/s41598-025-01289-4)

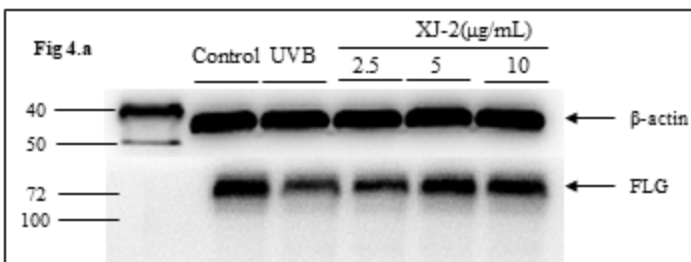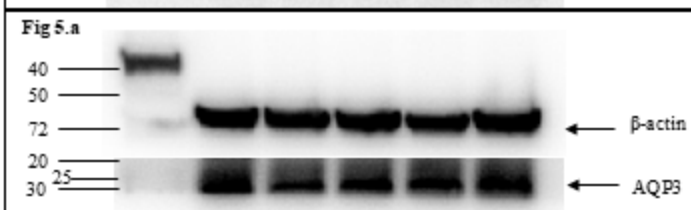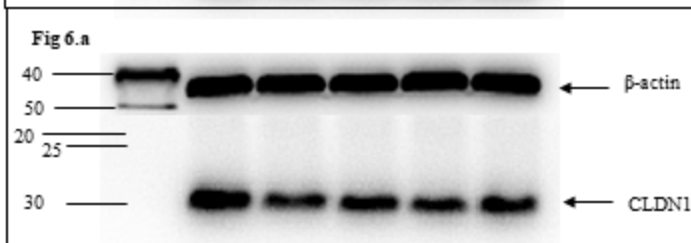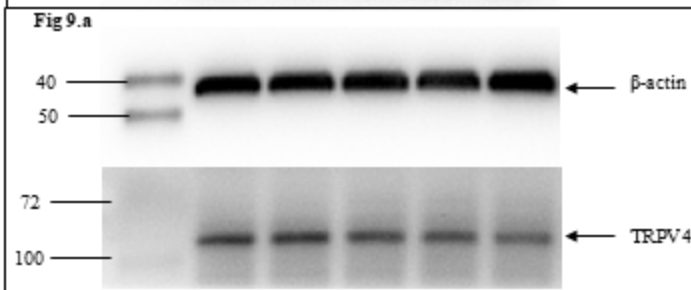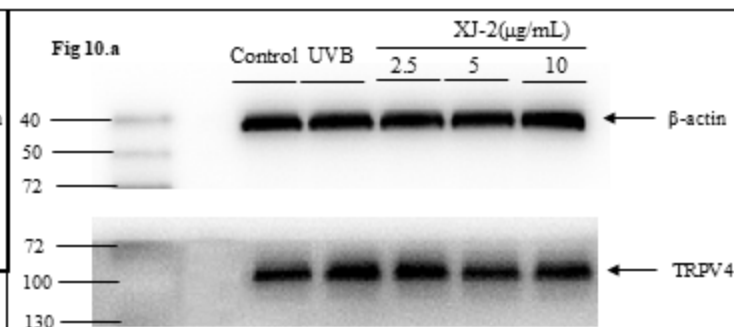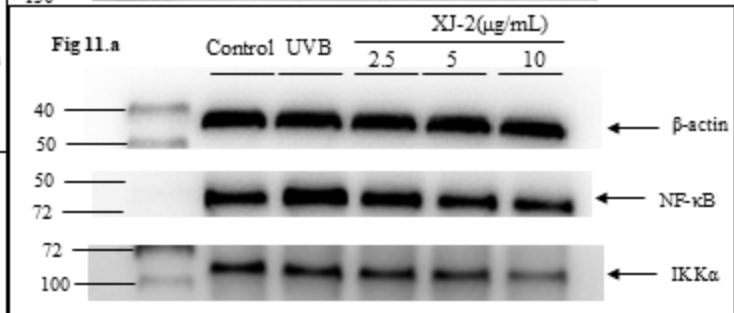

Fig.4 FLG

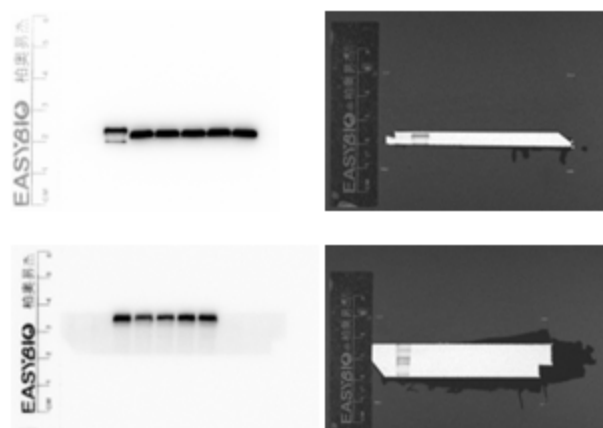

Fig.6 CLDN1

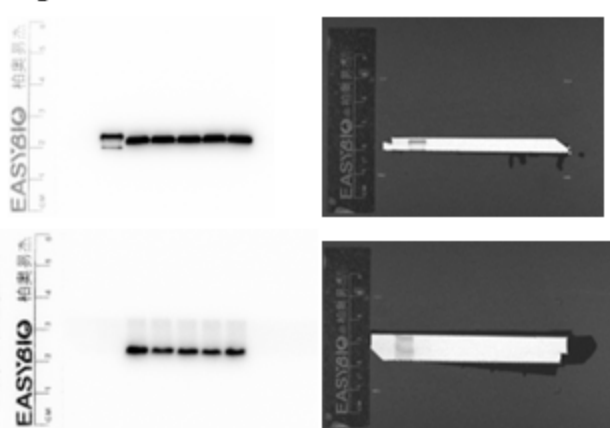

Fig.5 AQP-3

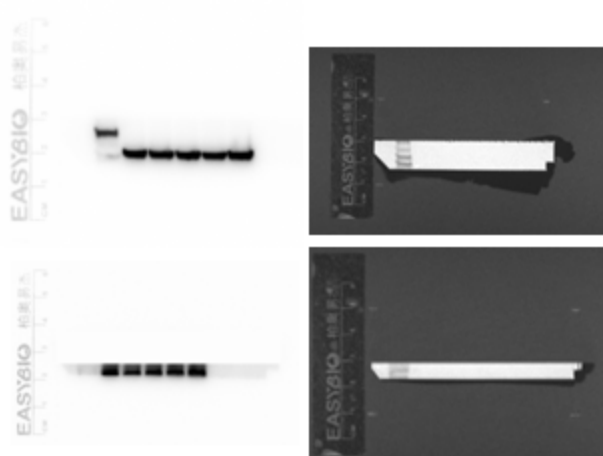

Fig.9 TRPV4

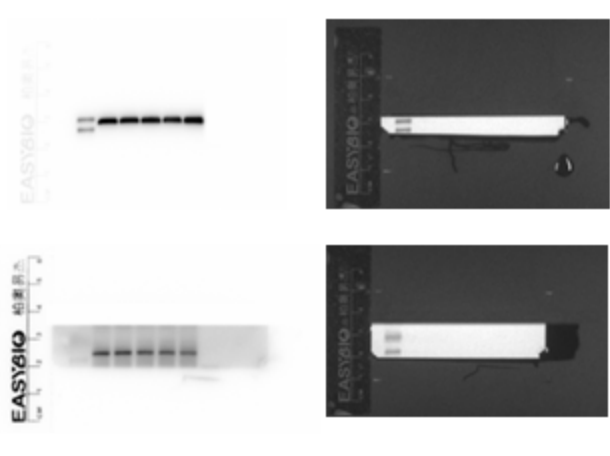

Fig.10 TRPV4

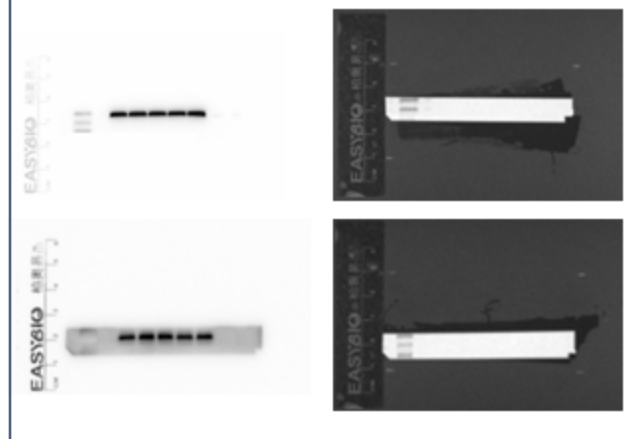

Fig.11 NF- $\kappa$ B/IKK $\alpha$

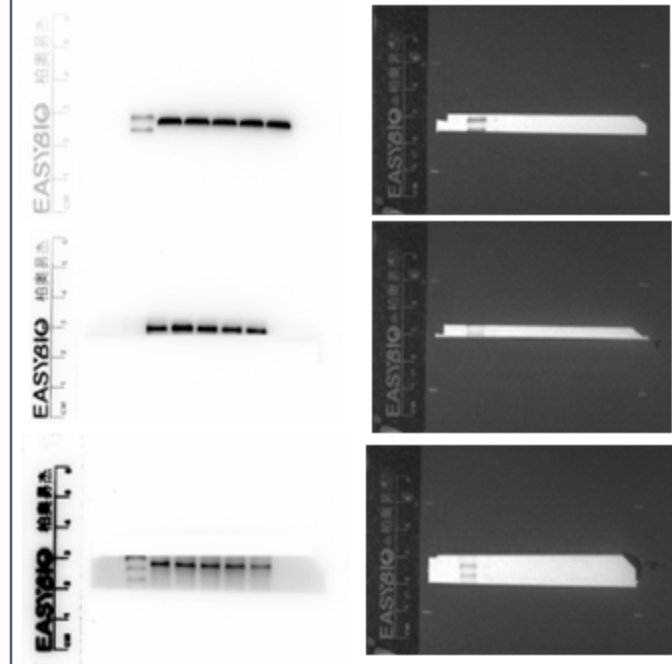

Supplement: Supplementary file 1 — Supplementary Material 1 [file 41598_2025_1289_MOESM1_ESM.pdf]
